# Prevalence and predictors of tuberculosis among HIV patients who completed isoniazid preventive therapy (IPT) at Reach out Mbuya community health initiative

**DOI:** 10.1038/s41598-023-44649-8

**Published:** 2023-10-16

**Authors:** Wycliff Sekayi, Edity Namyalo, Josephine Namayanja, Joseph M. Kungu

**Affiliations:** College of Veterinary Medicine, Animal Resources and Biosecurity, Makerere University, P.O. Box 7062, Kampala, Uganda

**Keywords:** Diseases, Health care, Risk factors

## Abstract

Tuberculosis (TB) continues to be the leading cause of morbidity and mortality among human immunodeficiency virus (HIV) infected individuals in Sub Saharan Africa, including Uganda. Isoniazid prophylaxis therapy (IPT) is a major public health intervention to limit tuberculosis disease among people living with HIV. However, there is limited information about the influence of IPT on TB disease incidence and its associated risk factors among HIV positive patients in Uganda especially at Reach out-Mbuya community health initiative hence the study. A cross sectional study was conducted among HIV positive adult patients who completed a 6-months long daily treatment of Isoniazid preventive therapy. Sputum samples and urine samples were collected and analysed using Gene Xpert and lateral flow urine Lipoarabinomannan (LF-LAM) tests respectively for presence of Tuberculosis disease. Data analysis was performed using STATA (version 14). Bivariate and multivariate logistic regression were performed to assess the risk factors associated with tuberculosis among the study population and significance estimated at 95% confidence level. A total of 103 HIV positive adults was studied. The mean age of the participants was 42.1 (10.5) and median age was 43 (IQR = 16). The prevalence of tuberculosis disease among HIV positive adult patients who completed Isoniazid preventive therapy was 5.8% (6/103). Counselling, the only significant factor, reduced the likelihood of occurrence of TB disease among HIV patients on IPT treatment (aOR:0.028, P-value < 0.001). A low prevalence of TB disease was observed among HIV patients on IPT treatment. Counselling is a protective factor of TB disease among HIV patients on IPT treatment.

## Introduction

Worldwide, tuberculosis (TB) is considered the leading cause of premature death among HIV/AIDS patients^1,2^. Tuberculosis is caused by *Mycobacterium tuberculosis* and mainly affects the lungs but it can also affect other body parts such as the brain, kidneys, or spine. HIV coinfection is the prime risk factor for developing active TB in the high-burden settings. This in turn increases the susceptibility to primary infection or reinfection and the risk of TB reactivation for patients with latent TB^1,3^. *Mycobacterium tuberculosis* also has a negative effect on the immune response to HIV, accelerating the progression from HIV infections to AIDS^4^. Furthermore, the risk of developing TB among people living with HIV (PLHIVs) is 18 times higher than the general population^5^.

According to WHO, up to 10.6 million people were estimated to be affected with TB worldwide in 2021, with up to 1.6 million TB deaths recorded including 187,000 HIV-positive people^2^. According to the report, Sub-Saharan Africa in 2017 was home to 70% of all people living with HIV/TB co-infection in the world. Uganda is among the 30 high TB burden countries and these countries accounted for 87% of new TB cases^2^.

The WHO recommends Isoniazid preventive therapy, intensified case finding and infection control (three ‘I’s) as the main strategies in the prevention of TB among people living with HIV^6^.

Studies have shown that IPT can significantly lower TB incidence among PLHIV after completion of the 6 month period of treatment^7^. However, uptake of IPT has been relatively low in most developing countries including Uganda^2,8^.

Information on enrolment, retention, challenges faced, loss to follow up, completion and prevalence of TB during the treatment period has been documented^5,9,10^. However, there are hardly any studies documenting prevalence of TB among PLHIV who have completed IPT therapy and its associated predisposing factors, hence the study.

## Methods

### Study design

A cross sectional study was conducted among PLHIV above 18 years who completed Isoniazid preventive therapy and had their clinic appointments between July and September, 2022. The study was carried out at Reach out Mbuya community health initiative in Nakawa division, Kampala district, central Uganda. Reach out Mbuya is a community-based non-governmental organization providing a unique model of care to PLHIV and where necessary their families; taking care of the medical, social, emotional and economical aspect of their lives to foster dignified living. The organisation started with 14 HIV positive clients in 2001, and currently serves ≥ 7000 annually.

### Eligibility criteria

All the people living with HIV/AIDS above 18 years, on Antiretroviral treatment (ART), TB disease negative, with documented records of Isoniazid preventive therapy completion and had consented were included in the study.

All potential participants with comorbidities that could compromise with their immunity, those who experienced side effects while on ART, and those patients who did not complete treatment due to personal or medical reasons were excluded from the study.

### Sample size estimation and sampling procedure

The sample used in the study was estimated using the formula for sample size calculation for a known population as follows^11^;

$$ {\text{n}} = \frac{1}{{\left( {1/{\text{n}}_{0} + 1/{\text{N}}} \right)}} $$

where,

$$ {\text{n}}_{0} = \frac{{{\text{Z}}^{2} {\text{P}}\left( {1 - {\text{P}}} \right)}}{{{\text{D}}^{2} }} $$

Sample prevalence (P) of 7.2% from a previous study in Uganda done by Moore et al.^12^, a margin of error (5%), (D = 0.05) and 95% level of confidence (Z = 1.96) and a known population (N) of 1000 patients were used to calculate sample size of 93 participants. To increase precision of the study, a total of 103 participants was selected using simple random sampling technique from a list of all patients who had completed IPT and had their clinic appointments in the July–September quarter, 2022.

### Data collection

A questionnaire was entered in Kobocollect toolbox software and used to capture participants’ demographic data and data assessing potential predisposing factors for TB in people living with HIV/AIDS. Such factors included; patient smoking, ability to disclose their HIV status, having awareness of isoniazid therapy as a preventive measure against TB, experienced side effects during isoniazid treatment period (allergic reactions, drowsiness), considered taking ART and isoniazid therapy burdensome (with regards to different types and quantities of pills to be taken), experienced stigma when observed by public that they were on treatment and whether they attended counselling sessions. The patients were asked to produce sputum samples into a clean, sterile, leak proof and labelled sputum cup for gene xpert test. Those who were not able to produce the sputum were requested to provide urine in a leak proof, screw capped labelled urine sample container.

### Analysis of samples

#### Gene Xpert

The sputum samples were tested using Gene Xpert test to detect presence of Mycobaterium Tuberculosis with a Cepheid machine developed by Cepheid International^13^. The test is a cartridge based nucleic acid amplification rapid test, highly sensitive and specific for *Mycobacterium tuberculosis* as well as Rifampicin resistance detection^14^.

#### Lateral flow urine lipoarabinomannan (LF-LAM) assay

The urine samples from the people who were not able to produce sputum were tested using a lateral flow urine lipoarabinomannan (LF-LAM) commonly known as urine LAM. The test which was developed by WHO employs highly purified antibodies specific for the major polysaccharide antigen of the Genus *Mycobacterium* in urine^15^.

### Data analysis

The data collected using Kobo toolbox was extracted, cleaned in Microsoft excel and analysed using STATA (version 14) statistical package. Logistic regression analysis was performed to determine the relationship between potential predisposing factors and prevalence of TB among HIV patients.

### Ethics approval and consent to participate

This study was approved by Mulago Hospital Research Ethics Committee (protocol MHREC 2370). Informed consent was obtained from all subjects and/or their legal guardian(s) before their participation in the study. Participants' details were kept confidential by using special data codes. All methods were performed in accordance with the relevant guidelines and regulations of the journal.

## Results

### Socio-demographics of the participants

A total of 103 PLHIV were included in the study. Majority of the participants were female 62 (60.2%). The mean age of the participants was 42.1 (SD = 10.5) and median age was 43 (IQR = 16). Most of the participants 54 (52.43%) were urban dwellers, with up to 37 (35.7%) having attained secondary level of education. Up to 58 (56.3%) participants were married, 59 (57.3%) were employed and 33(33.0%) were Catholics. Details are shown in Table 1.

**Table 1:** Frequency distribution of social-demographic characteristics.

Characteristic	Frequency	Percentage (95% CI)
Age		
≤ 30	18	17.5 (11.2–26.2)
31–50	64	62.1 (52.3–71.1)
≥ 51	24	20.4 (13.6–62.0)
Gender		
Male	41	39.8 (30.7–49.7)
Female	62	60.2 (50.3–69.3)
Residence		
Urban	52	52.4 (42.7–62.0)
Sub-urban	34	33.0 (24.5–42.8)
Rural	15	14.6 (8.9–22.9)
Education level		
None	11	10.7 (6.0–18.4)
Primary	28	27.2 (19.4–36.7)
Secondary	37	35.9 (27.1–45.8)
Tertiary	27	26.2 (18.5–35.7)
Marital status		
Divorced/separated	22	21.4 (14.4–30.5)
Lives with partner	58	56.3 (46.5–65.7)
Single	11	10.7 (5.9–18,4)
Widowed	12	11.6 (6.7–19.6)
Employment		
Employed	59	57.3 (47.4–66.6)
Unemployed	44	42.7 (33.4–52.6)
Religion		
Anglican	28	27.2 (19.4–36.7)
Catholic	33	33.0 (24.5–42.8)
Moslem	23	22.3 (15.2–31.5)
Pentecost	18	17.5 (11.2–26.2)

### Prevalence of tuberculosis

The overall prevalence of TB among the study participants was 5.8%. Out of the 65 patients tested by Gene Xpert, only 3 (4.6%) were TB positive, while out of 38 patients tested by TF-LAM, 3 (7.9%) were positive as shown in Table 2.

**Table 2:** Prevalence of tuberculosis.

Test	Gene Xpert	TF LAM	Total
TB negative	62	35	97
TB positive	03	03	06
Prevalence (%)	4.6 (0.5–9.7)	7.9 (0.7–16.5)	5.8 (1.3–10.3)

### Predisposing factors of TB in PLHIV who completed IPT

Bivariate and multivariate logistic regression were performed to assess the association between TB prevalence (dependent variable) and various predisposing factors (independent variables).

Bivariate analysis showed that being married (living with a partner) (OR = 0.171, P = 0.014) and receiving counselling (OR = 0.013, P = 0.001) had a relationship with TB prevalence among HIV patients (Table 3).

**Table 3:** Bivariate logistic regression of predisposing factors with TB in HIV patients.

Characteristic	Number (%)	TB (%)	Odds ratio (95%CI)	P-value
Age				
≤ 30	18 (17.1)	1(5.6)	0.559(0.046–6.727)	0.647
31–50	64 (62.5)	3 (4.7)	0.467(0.073–3.007)	0.423
≥ 51	24 (20.4)	2 (9.5)	Reference	
Gender				
Female	62 (60.2)	02 (3.2)	Reference	
Male	41 (39.8)	04 (9.8)	1.980 (0.580–6.755)	0.275
Religion				
Catholic	34 (33.0)	04 (11.8)	Reference	
Anglican	28 (27.2)	02 (7.1)	0.714 (0.179–2.847)	0.633
Moslems	23 (22.3)	00 (00)	1.143 (0.129–8.436)	0.968
Pentecost	18 (17.5)	00 (00)	2.234 (0.322–15.515	0.416
Marital status				
Divorced/separated	22 (21.4)	01 (4.5)	Reference	
Lives with partner	28 (26.3)	02 (3.4)	0.171 (0.045–0.697)	**0.014**
Single	11 (10.7)	01 (9.0)	1.143 (0.124–8.512)	0.896
Widowed	12 (11.6)	02 (16.7)	2.047 (0.341–12.285	0.433
Education				
None	11 (10.7)	00 (00)	Reference	
Tertiary	27 (26.2)	00 (00)	1.835 (0.192–17.494)	0.598
Secondary	37 (35.9)	03 (8.1)	0.877 (0.102–7.568)	0.905
Primary	28 (27.2)	03 (10.7)	2.224 (0.334–14.385	0.401
Residence				
Rural	15 (14.6	00 (00)	Reference	
Urban	54 (52.4)	06 (11)	1.758 (0.728–4.242)	0.209
Sub-urban	34 (33)	00 (00)	0.843 (0.428–1.659)	0.621
Employment				
Unemployed	44 (42.7)	04 (9.1)	Reference	
Employed	59 (57.3)	02 (3.4)	3.326 (0.824–13.417)	**0.091**
Alcohol use				
No	56 (54.4)	00 (00)	Reference	
Yes	47 (45.6)	06 (10.7)	2.402 (0.421–13.700)	0.324
Smoking				
Non-smoker	96 (93.2)	05 (5.2)	Reference	
Smoker	07 (6.8)	01 (14.3)	1.648 (0.183–14.876)	0.656
Disclosure				
Not disclosed	27 (26.2)	04 (14.8)	Reference	
Disclosed	76 (73.8)	02 (2.6)	0.181 (0.335–4.164)	0.795
Awareness of IPT as preventive measure against TB				
No	24 (23.3)	05 (20.8)	Reference	
Yes	79 (76.7)	01 (1.3)	0.455 (0.109–1.905)	0.482
Side effects				
Not affected	57 (55.3)	00 (00)	Reference	
Affected	46 (44.7)	06 (13)	1.068 (0.315–3.618)	0.916
Pill burden				
Not burdensome	61 (59.2)	00 (00)	Reference	
Burdensome	42 (40.8)	06 (14.3)	1.100 (0.220–5.503)	0.908
Stigma				
Not stigmatized	70 (68.0)	00 (00)	Reference	
Stigmatized	33 (32.0)	06 (18.2)	1.029 (0.170–3.123)	0.670
Adherence counselling				
Not counselled	41 (39.8)	04 (9.8)	Reference	
Counselled	62 (60.2)	02 (3.2)	0.013 (0.01–0.175)	**0.001**

Significant values are in bold.

Factors with P-values ≤ 0.1 such as marital status, employment and adherence to counselling were included in a model for multivariate logistic stepwise regression analysis. It was observed that only counselling was the only significant factor associated with prevalence of TB in HIV patients (aOR:0.028, P-value < 0.001, 95% CI 0.0041–0.1924) (Table 4).

**Table 4:** Multivariate logistic regression of predisposing factors with TB in HIV patients.

Variable	aOR	Z	P-value	95% CI
Counselling	0.028	− 3.64	< 0.001*	0.0041–0.1924
Marital status	0.855	− 0.37	0.709	0.3756–1.9455

Significant values are in bold.

## Discussion

Tuberculosis continues to be the leading cause of mortality among PLHIVs in Sub-saharan Africa including Uganda. The risk of developing TB among people living with HIV (PLHIVs) is higher than the general population^5^. Therefore, WHO strongly recommends the use of Isoniazid preventive therapy to TB among PLHIVs to fight TB incidences in addition to infection control and active case finding^16^.

A number of studies have reported that IPT significantly reduces incidence of TB among PLHIV after completion of the 6 month period of treatment^17–19^. This study recorded a TB prevalence of 5.8% among PLHIV who had completed the 6 months period of IPT treatment, which is lower than 8.1% and 9.0% reported respectively in studies conducted in Ethiopia^13,14^. This difference might be attributed to the different study design and sampling techniques used. Other studies reported similar findings of lower incidence rates among PLHIV that completed IPT therapy than those who were not IPT therapy^17–19^.

The study observed that females had higher completion rates of IPT as compared to their male counterparts. This is in agreement with a study in Butebo, Uganda which reported that females had a higher uptake of IPT (66%) as compared to males (33.8%) in 2021^8^.

Although TB disease was higher in males (9.8%) than in females (3.2%) in this study, gender was not an important predictor of TB disease prevalence as similarly reported in previous studies^8,16^.

It was observed in this study that adherence to counselling was an important predictor that reduced the likelihood of TB disease occurence. This concurs with previous studies in Ethiopia and Uganda that pre-counselling of HIV clients prior to enrolment of IPT significantly improved IPT uptake and completion hence preventing active TB^20^.

Although this study provides baseline evidence regarding prevalence and predisposing factors of active TB among PLHIV on IPT, the findings can not be used to affirm that administration of IPT to PLHIV effectively reduced occurrence of TB. This is because a control group of PLHIV who had not completed IPT was not included for comparative analysis. The prevalence of active TB before and after IPT was also not determined.

## Conclusion

The study observed a considerably low prevalence of TB among PLHIV that had undergone IPT.

Counselling was noted to be a significant predictor of prevention of active TB among HIV patients. Therefore, in addition to the recommended 3Is, consistent counselling (pre and during treatment) should be emphasized in ensuring effectiveness of the IPT among PLHIV (Supplementary Information).

### Supplementary Information

Supplementary Information.

## Data Availability

The datasets used and analysed during the current study are available as supplementary files.

## Abbreviations

IPT: Isoniazid preventive therapy
HIV: Human immunodeficiency virus
AIDS: Acquired immunodeficiency syndrome
PLHIV: People living with human immunodeficiency virus
WHO: World Health Organisation
ART: Antiretroviral therapy
CI: Confidence interval
aOR: Adjusted odds ratio
TF LAM: Lateral flow urine lipoarabinomannan

## References

[1] Komrower, D. & Thillai, M. Tuberculosis and HIV co-infection. In *Clin Tuberc A Pract Handb.* 157–70 (2015).

[2] WHO. Global tuberculosis report (2022).

[3] Mebratu W, Wedajo S, Mohammed S, Endawkie A, Damtew Y (2022). Prevalence and associated factors of tuberculosis among isoniazid users and non-users of HIV patients in Dessie, Ethiopia. Sci. Rep..

[4] Bruchfeld, J., Correia-neves, M. & Ka, G. *Tuberculosis and HIV Coinfection*. 1–15 (2015). 10.1101/cshperspect.a017871 PMC4484961 25722472

[5] WHO. Global tuberculosis report. (2020). https://www.who.int/publications/i/item/9789240013131.

[6] Charles MK, Lindegren ML, Wester CW, Blevins M, Sterling TR, Dung NT (2016). Implementation of tuberculosis intensive case finding, isoniazid preventive therapy, and infection control (“Three I’s”) and HIV-tuberculosis service integration in lower income countries. PLoS ONE..

[7] Robert M, Todd J, Ngowi BJ, Msuya SE, Ramadhani A, Sambu V (2020). Determinants of isoniazid preventive therapy completion among people living with HIV attending care and treatment clinics from 2013 to 2017 in Dar es Salaam Region, Tanzania. A cross-sectional analytical study. BMC Infect. Dis..

[8] Oonyu L, Kang S, Konlan KD, Ae KY (2022). Isoniazid preventive therapy for tuberculosis in people living with HIV: A cross sectional study in Butebo, Uganda. Infect. Chemother..

[9] Tiruneh, G., Getahun, A. & Adeba, E. Assessing the impact of isoniazid preventive therapy (IPT) on tuberculosis incidence and predictors of tuberculosis among adult patients enrolled on ART in Nekemte Town, Western Ethiopia: A retrospective cohort study. *Interdiscip. Perspect. Infect. Dis.* **2019** (2019). 10.1155/2019/1413427 PMC6521313 31186628

[10] Beshaw MA, Balcha SA, Lakew AM (2021). Effect of isoniazid prophylaxis therapy on the prevention of tuberculosis incidence and associated factors among HIV infected individuals in northwest Ethiopia: Retrospective cohort study. HIV/AIDS Res. Palliat. Care..

[11] Dohoo, I., Martin, W. & Stryhn, H. *Methods in Epidemiologic Research* 48–54 (2012).

[12] Moore D, Liechty C, Ekwaru P, Were W, Mwima G, Solberg P (2007). Prevalence, incidence and mortality associated with tuberculosis in HIV-infected patients initiating antiretroviral therapy in rural Uganda. Aids..

[13] Cepheid International. Cepheid. A better way. (2011). https://www.cepheid.com/en-GB.

[14] CDC. TB Diagnostic tool: Xpert MTB/RIF assay fact sheet. (2016). 1. http://www.cdc.gov.

[15] WHO. Lateral flow urine lipoarabinomannan assay (LF-LAM) for diagnosis of active tuberculosis in people living with HIV, 2019 update. (2019). https://www.who.int/publications. 10.1002/14651858.CD011420.pub3 PMC6802713 31633805

[16] Charles MK, Lindegren ML, Wester CW, Blevins M, Sterling TR, Dung NT (2016). Implementation of tuberculosis intensive case finding, isoniazid preventive therapy, and infection control (“Three I’s”) and HIV-tuberculosis service integration in lower income countries. PLOS ONE..

[17] Maokola WM, Ngowi BJ, Mahande MJ, Todd J, Robert M, Msuya SE (2021). Impact of Isoniazid Preventive Therapy on Tuberculosis incidence among people living with HIV: A secondary data analysis using Inverse Probability Weighting of individuals attending HIV care and treatment clinics in Tanzania. PLoS ONE..

[18] Legese H, Degefa H, Gebrewahd A, Gebremedhin H (2020). Utilization of isoniazid prophylaxis therapy and its associated factors among HIV positive clients taking antiretroviral therapy at Fre Semaetat primary hospital, Hawzien districts, Tigrai, Northern Ethiopia. Trop. Dis. Travel. Med. Vaccines..

[19] Sabasaba A, Mwambi H, Somi G, Ramadhani A, Mahande MJ (2019). Effect of isoniazid preventive therapy on tuberculosis incidence and associated risk factors among HIV infected adults in Tanzania: A retrospective cohort study. BMC Infect. Dis..

[20] Mebratu W, Wedajo S, Mohammed S, Endawkie A (2022). Prevalence and associated factors of tuberculosis among isoniazid users and non-users of HIV patients in Dessie, Ethiopia Statistical Package for Social Sciences. Sci. Rep..

